# René Girard and the Mimetic Nature of Eating Disorders

**DOI:** 10.1007/s11013-018-9574-y

**Published:** 2018-03-07

**Authors:** Mattias Strand

**Affiliations:** 1 Stockholm Centre for Eating Disorders, Wollmar Yxkullsgatan 27B, 118 50 Stockholm, Sweden; 2 Department of Clinical Neuroscience, Karolinska Institutet, Stockholm, Sweden

**Keywords:** René Girard, Eating disorders, Anorexia nervosa, Bulimia nervosa

## Abstract

French historian and literary critic René Girard (1923–2015), most widely known for the concepts of mimetic desire and scapegoating, also engaged in the discussion of the surge of eating disorders in his 1996 essay *Eating Disorders and Mimetic Desire*. This article explores Girard’s ideas on the mimetic nature and origin of eating disorders from a clinical psychiatric perspective and contextualizes them within the field of eating disorders research as well as in relation to broader psychological, sociological and anthropological models of social comparison and non-consumption. Three main themes in Girard’s thinking on the topic of eating disorders are identified and explored: the ‘end of prohibitions’ as a driving force in the emergence of eating disorders, eating disorders as a phenomenon specific to modernity, and the significance of ‘conspicuous non-consumption’ in the emergence of eating disorders.

## Introduction

In the essay *Eating Disorders and Mimetic Desire*, originally published in 1996, French historian and literary critic René Girard (1923–2015) takes on the topic of eating disorders (EDs)—his interest apparently sparked by seeing his university students struggle with restrictive eating. The essay was later published in book format with the addition of a conversation on the topic with anthropologist Mark Anspach and journalist Laurence Tacou (Girard, Anspach, and Tacou 2013). The aim of the present paper is to contextualize Girard’s ideas within contemporary research on EDs, body image, and peer influences in order to examine whether they can inform the current thinking on the mechanisms behind the development of EDs.

The *Diagnostic and Statistical Manual of Mental Illness, 5th Edition* (DSM-5; American Psychiatric Association 2013) distinguishes between three primary EDs: anorexia nervosa (AN), bulimia nervosa (BN), and binge eating disorder (BED). AN is characterized by restriction of energy intake leading to a significantly low body weight, intense fear of weight gain (so called ‘weight phobia’), and a disturbed experience of one’s own body weight or shape. BN is characterized by recurrent episodes of binge eating, inappropriate compensatory behaviors to prevent weight gain (such as vomiting, misuse of laxatives, or excessive exercise), and excessive emphasis on weight or shape in self-evaluation. BED is characterized by recurrent episodes of binge eating without compensatory behaviors. AN has the highest mortality rate of any mental illness, at approximately 5% annually (Arcelus et al. 2011). AN and BN typically debut in adolescence or young adulthood (Hudson et al. 2007), whereas little is known about the development of BED (American Psychiatric Association 2013). Diagnostic crossover between ED diagnoses is common over time (Eddy et al. 2008). The 12-month prevalence is estimated to 0.4% for AN and to 1.0–1.5% for BN in young females; 12-month prevalence of BED among adults is estimated to 1.6% for females and 0.8% for males (American Psychiatric Association 2013). However, disordered eating in the absence of a full ED is significantly more common: in adolescence, about 24% of girls and 16% of boys report disordered eating symptoms (Hautala et al. 2008).

Although—as will be discussed below—opinions differ as to whether EDs are modern phenomena or if they have actually been present throughout history, there is reason to believe that the prevalence of AN has increased during the 20th century (Theander 1970; Russell and Treasure 1989; Keel and Klump 2003). BN was first recognized as a clinical entity in 1979 (Russell 1979), and BED, although having been described in the 1950s (Stunkard 1959), was not officially accepted as a diagnosis in its own right until the launch of DSM-5 in 2013 (American Psychiatric Association 2013).

## René Girard on Eating Disorders

René Girard is most widely known for the concepts of *mimetic desire* and *scapegoating*, which he developed in a large number of books over the years. In his work, Girard points to the importance of imitation in human behavior, invoking the Greek word *mimesis*—a term that carries many possible meanings, including ‘representation’ and ‘imitation’. Girard argues that human desire is also mimetic in this imitative sense: we principally experience desire based on seeing other people desire something, rather than on some intrinsic value of the desired object itself. Desire becomes competitive not because there is necessarily a scarcity of the object in question, but because desire is essentially related to the wants of others. In Girard’s view, this phenomenon is ever-present. Girard argues that, contrary to the common belief that conflict stems from irreconcilable differences, it is our ultimate *sameness* in desire that creates and perpetuates antagonism. Rivalry actually effaces differences and promotes a spiral of imitation.

A direct consequence of this mimetic structure is escalating violence. In his 1972 book *Violence and the Sacred*, Girard refers to blood feuds to make his point (Girard 2013). In a blood feud, vengeance becomes an infinitely repetitive process whereby bloodshed is met with more bloodshed, to a point where the original crime fades from visibility and loses all importance. The object of the feud becomes irrelevant; what matters is the retributive imitation. As more and more people are drawn into this mimetic whirlpool, the situation eventually becomes disastrous for the community.

In order to circumvent this scenario a scapegoat is created, something Girard refers to as a more or less automatic “braking mechanism” (Girard 2013:74). The mimetic rivals hereby find a way to neutralize conflict by directing their violence towards a third party, thus inventing a common enemy that can be sacrificed in order to purify the society and reinstate peace. Importantly, for the scapegoat to be able to perform this curbing of violence it must exist in a state of ambiguous in-betweenness—it must be at once part of the community and alien to it. Should the designated scapegoat be too distant, the violence directed towards it will lack neutralizing meaning for the feuding parties; should it be too close, the pendulum motion of mimetic vengeance will be upheld. Typically, viable scapegoats have been marginalized groups in society; however, Girard also points to the fact that *kings* readily fill the same ambiguous in-between requirement.

As commentator Christian Fleming notices, René Girard was not a philosopher in the strict sense and his work does not truly belong to any of the main philosophical currents of the twentieth century (Fleming 2004). Needless to say, there is a large and heterogeneous body of literature on the subject of mimesis as representation, imitation, mimicry, etc. (see for example Adorno 1997; Auerbach 1953; Benjamin 2005; Derrida 1981; Taussig 1993), but while many of these works can inform the study of Girardian concepts, they are not necessarily thematically coherent. For literary critic Erich Auerbach, the concept of mimesis described how representation of reality in literature mirrors social conventions and developments in the epoch in which it was written. Theodor Adorno and Max Horkheimer of the Frankfurt School saw mimesis as a historical phenomenon of adaptation to a harsh natural world—much like mimicry in plants and animals. As civilization progresses, this type of rudimentary mimesis-by-necessity is supposedly replaced by institutionalized forms of control, first by magic and, in our days, by rationality and man’s dominance over nature. Critical theorist Walter Benjamin introduced the idea of a more benevolent *mimetic faculty* in mankind: an ability to form and perceive similarities recognizable in most human activities, from imitative child’s play to human language. This concept was later developed by anthropologist Michael Taussig, who identified mimetic representations of artefacts from Western culture in the cosmology of the Cuna people in Panama. For Taussig, mimesis appears to resemble the concepts of *assimilation* or *appropriation* whereby the copy also affects the original. In a somewhat similar vein, philosopher Jacques Derrida described mimesis as central to the concept of deconstruction. In Derrida’s work, mimetic repetition does not merely produce likeness but, in fact, something new, thereby deconstructing the notion of original and copy and conceptualizing mimesis as an unreducible something-in-itself without an external reference. The Girardian take on mimesis, in contrast, is not primarily concerned with aesthetics, linguistics or intertextuality. For Girard, mimesis forms a fundamental fabric of human society. His focus on mimetic *rivalry* takes on an archaic quality, with the mechanism of scapegoating serving almost as a myth of creation and with mimetic violence looming apocalyptically in the shadows. Whereas for Adorno and Horkheimer, mimesis belongs to a primordial past, Girard recognizes mimetic phenomena constantly lurking beneath the mundane surface of everyday life.

There are contemporary thinkers that share Girard’s interest in communal acts of violence; for example, literary theorist Kenneth Burke has written extensively about how communities enact a purging ‘drama’ of expulsion and victimization in times of societal transformation (Burke 1966). The Girardian scapegoat does to a certain extent also resemble the notion of the *abject*—i.e., that which used to be a subject but has been transformed into an ambiguously repulsive object—in the work of philosopher and psychoanalyst Julia Kristeva (Kristeva 1982; see also Warin 2010). What sets Girard apart is the fact that he is first and foremost concerned with *violence* in its most archetypal and yet ever-present forms—one commentator notes, paraphrasing Lacan, that for Girard, ”the unconscious is structured like a lynching” (Sollers 1986:191). It must, however, be noted that this violence is at once purging and generative. Important to the discussion below, Girard views many contemporary societal rituals and institutions as instances of formalized scapegoating that has emerged more or less unconsciously in order to safeguard against violence in its much more catastrophic mimetic form.

Girard’s approach to the surge of eating disorders in *Eating Disorders and Mimetic Desire* will be examined in detail below; however, a brief introductory overview is in order. In opposition to the focus on dysfunctional parenting favored at the time, Girard argues that EDs stem primarily from mimetic comparison. Just as mimesis may turn largely anything into a precious object of desire, so it elevates the quest for slenderness to a predominant rivalry of our time, setting in motion bodily pursuits that risk spiraling out of control. Girard argues that contemporary theories on the origin of EDs—psychoanalytic, system oriented, feminist, etc.—all overlook the mimetic properties of these conditions, or at least put too little emphasis on them. He writes: “The problem is not that these eating disorders are too complex for our current systems of interpretation […]. The problem is that they are too simple, too readily intelligible” (p. 3).^1^ To Girard, EDs are—or should be—easily comprehensible because they mirror an ever-present communal desire: “The compulsive dieters really want to be thin and most of us are secretly aware of this because most of us also want to be thin. All our convoluted systems of explanation […] are floundering on this ridiculous but irrefutable evidence.” (p. 3) In speaking about desire, Girard correctly notes that the term anorexia—i.e., ‘lack of appetite’—is misleading: “Contrary to what the etymology of the word deceptively suggests, the anorexic^2^ has an appetite. She still wants to eat just as much as we do and much more, because she is hungrier than we are. Some anorexic patients fear that if they ate a single bite, they would never stop eating.” (p. 4)

Furthermore, Girard argues that the subdivision of EDs into separate clinical entities such as AN or BN is superficial—again, for Girard this is anchored in common experiences:

Why distrust the distinction between symptoms as radically opposed as those of anorexia and bulimia? Because we live in a world where eating too much and not eating enough are opposite but inseparable ways of coping with the slenderness imperative that dominates our collective imaginations. Most of us oscillate all our lives between attenuated forms of these pathologies. (p. 1)

Girard also criticizes the popular contemporary notion of comparing the clinical EDs of today to the ascetic ‘holy fasting’ of antiquity and the medieval period (Bell 1987; Vandereycken and van Deth 1994). Discarding this view, he describes AN as the archetypical modern syndrome, “unleashed by the end of prohibitions” (p. 9) rather than by religious and social restrictions.

In Girard’s view, the widespread idea that perpetual scarceness of food in centuries past led to a plump, buxom beauty standard associated with a well-fed elite is a distortion of history. Dismissing this rotund ideal as a myth, he argues that in the old days people simply did not give that much thought to weight and body shape. Instead, it is our contemporary society that constitutes the historical exception in our passion for thinness—or, one might argue, for whatever body ideal currently in fashion. Unwilling to accept this, we clamp a “fatness imperative” upon history as “a crude projection of our own obsession with food, an obvious maneuver to deny our own singularity” (p. 15). For Girard, EDs are genuinely modern phenomena.

For the sake of this article, the discussion on EDs raised by Girard can be summarized in three main questions. First, is there support for Girard’s view of the etiology behind EDs as “too simple”, in contrast to unnecessarily complex systemic or psychodynamic views? Secondly, are EDs time-specific phenomena, emerging as a result of the particular historical and societal circumstances of our time, or have they been present throughout history? Thirdly, how can phenomena of ‘non-consumption’, such as restrictive eating, be construed as mimetic? These are, of course, extensive questions and any definitive answers cannot be expected to arise from this overview. Instead, the purpose of this article is to enter into dialogue with Girard’s views on the nature of EDs, to put them in a clinical context and to investigate whether his mimetic perspective can help elucidate these matters. In the first section, Girard’s notion of cultural change as a driving force behind EDs is discussed in relation to the conceptualizations of influential psychiatrists Hilde Bruch and Mara Selvini Palazzoli as well as in light of newer research on the impact of migration and acculturation. In the second section, Girard’s take on the historicity of EDs is discussed vis-à-vis contemporary views on religious asceticism as either premodern ED variants or as non-interrelated phenomena. In the third section, mimetic aspects of non-consumption are addressed in relation to sociologist Thorstein Veblen’s consumption model, social psychologist Leon Festinger’s social comparison theory and research on peer and media influences. Critical voices, such as anthropologists Megan Warin and Anna Lavis and sociologist Abigail Bray, are also discussed.

Whereas these three analytical sections deal with the explicit proposals of Girard’s conceptualization of EDs, it appears equally important to highlight certain aspects that are entirely absent from his argument. The undeniable gendering of mimetic forces in relation to consumption and conspicuousness is discussed in the third section. A fourth section, finally, highlights aspects that are directly related to the ideas raised by Girard but that are unfortunately neglected by his predominant focus on mimetic pursuit of thinness: the heterogeneous discourses of body image concerns in different populations and the occurrence of mimesis without rivalry. Here, the work of medical anthropologists such as Eileen Anderson-Fye, Emily Yates-Doerr, and Alexandra Brewis, is discussed.

The aim of this paper is to contextualize the ideas of René Girard within contemporary research on EDs, body image, and peer influences in order to examine whether they can inform the current thinking on the mechanisms behind the development of EDs; it is not, however, to ultimately decide whether he was right or wrong. What follows will largely be a critical analysis of Girard’s account of mimesis in EDs, highlighting both viable and less viable aspects of his writing on the topic. Although Girard’s view on EDs is, from a clinical point of view, largely that of an ‘outsider’, he was—and is—a widely respected figure within the fields of social and anthropological philosophy. As such, his writing on the surge of EDs deserves critical attention. Commenting on another current ‘epidemic’, that of childhood obesity, medical anthropologist Tina Moffat writes:

[C]ritical scholars must begin a dialogue with biomedical professionals. At this point in time, there appears to be little in the way of communication between these groups and no public acknowledgment by medical professionals of the critique; at the same time critical scholars have not fairly considered biomedical perspectives […]. For there to be dialogue, there must be some common ground, which means an acknowledgment on the part of critical scholars that there is a biological phenomenon occurring globally that may well have some dire health consequences. (Moffat 2010:13)

The same holds true for the field of ED research. Hopefully, critical analysis of Girard’s writing can be one pathway in stimulating such interdisciplinary dialogue. Girard’s concept of mimetic desire has occasionally been mentioned in the ED literature (Allison, Warin, and Bastiampillai 2014; Lavis 2016a, b) but to the best of my knowledge, it has not previously been subjected to a more exhaustive survey within the field. Here, I should emphasize that my perspective in writing this article is that of a clinical psychiatrist specializing in EDs and that although my account partly relies on sociological and anthropological research, I am not formally trained in either of these disciplines. Furthermore, even though some of the literature referenced here explicitly questions the dominant biomedical model of EDs, my aim is not primarily to defend or challenge any particular explanatory model in the vast array that does exist, but to engage with Girard’s writing on the topic from different perspectives in hopes of generating a fruitful discussion.

As noted above, Girard also proposes a *transdiagnostical* view of EDs, suggesting that AN, BN and BED have more in common than what separates them and that they are merely different expressions of the same essential underlying mechanisms. This idea has been the subject of much debate (see for example Birmingham, Touyz, and Harbottle 2009; Fairburn, Cooper, and Shafran 2003; Waller 2008) but is not as central to Girard’s argument on the origin of EDs as the three main themes raised above and will not be investigated further in this review.

## Cultural Change as a Driving Force

When Girard argues that EDs are “too readily intelligible” (p. 3), he does so in explicit opposition to the Ricœurian notion of *hermeneutics of suspicion* (see for example Ricœur 1970):

The search for hidden motivations is the alpha and omega, of course, of our modern culture. Our number one principle is that no human phenomenon is really what it seems to be. […] We automatically assume that social phenomena have little if anything to do with what is obvious in them, in this case the rejection of food. (p. 2)

In fact, to Girard, “[g]iven the topsy-turvy relationship of our culture to food, it is not the rise in EDs which is astonishing but the fact, rather, that so many people eat more or less normally.” (p. 5) There is no need for turning over stones in the search for driving mechanisms behind EDs; the one central feature of both AN and BN—the wish for slenderness—is, supposedly, shared by us all. Girard even compares this situation to Thomas Hobbes’s ‘war of all against all’ (Hobbes 1996), noting that “[t]he spirit of rivalry may triumph in the absence of any specific rival” (p. 13), imposing on contemporary society a perpetual battle against a non-descript enemy. This battle is simultaneously internalizing and externalizing: “Mimetic desire aims at the absolute slenderness of the radiant being some other person always is in our eyes but we ourselves never are, at least in our own eyes. To understand desire is to understand that its self-centeredness is undistinguishable from its other-centeredness.” (p. 8)

Here, Girard identifies two seemingly opposite forces at play. On the one hand, there are forces promoting consumption: the availability of inexpensive food in abundance, marketing and advertising, and, finally, what he sees as the collapse of religious and ethical restraints. On the other hand, slenderness and restrictiveness are marketed in much the same way. These mixed messages, Girard argues, creates a paradox that threatens to trap people in a sense of hopelessness: “No wonder if we are also the culture from which many people want to drop out, as a result of sheer exhaustion, and also, perhaps, of a peculiar kind of boredom.” (p. 17) Whereas the forces of capitalism would appear to be an easy and perhaps predictable target in this discussion, Girard describes the capitalist system as more of a secondary player, reactively adjusting rather than actively imposing:

The capitalist system is clever enough, no doubt, to adjust to the rage for thinness and it invents all sorts of products supposedly capable to help us in our battle against calories, but its own instinct runs the other way. It systematically favors consumption over abstinence and it certainly did not invent our dieting hysteria. (p. 3)

Instead, Girard explicitly attributes the rise of the modern EDs to cultural change, having let loose forces of mimesis that had previously been contained by various cultural, familial and religious prohibitions. While he is not in defense of cultural conservatism, Girard argues that the rejection of what may rightly have been seen as oppressive cultural edicts inevitably came with a price: increased mimetic rivalry, “the uninvited guest that no one ever expects” (p. 7). Authority may have been rejected; competitiveness still reigns supreme. Also, unlike earlier historical periods, in our time no culturally sanctioned ‘safety valve’ is available: “These forces could recreate unanimity only through collective scapegoating, which cannot really occur, fortunately, in our world, because our notion of the human person […] prevents the reestablishment of a community founded on unanimous violence.” (p. 9) In the absence of prohibition as well as of the counterweight mechanism of scapegoating, phenomena such as EDs occur.

From the 1930s and onwards, psychoanalytic models of the nature of EDs flourished, often with an emphasis of the ‘refusal’ of food as a sign of fear of ‘oral impregnation’ (see for example Waller, Kaufman, and Deutsch 1940). As psychiatrist Hilde Bruch noted, this idea was so persistent that it developed a pathognomonic significance in AN, so that “if fear of impregnation could be elicited from a patient, even mild degrees of weight loss or neurotic vomiting were diagnosed as representing anorexia nervosa, though the clinical picture was quite unspecific” (Bruch 1973:217). In the 1960s and 1970s, this focus shifted as Bruch and others within the psychodynamic community started favoring family therapy as the treatment of choice. However, unlike today, the reason for actively involving parents was that they were routinely seen as the cause of the ED. Like Girard, psychiatrist and family therapist Mara Selvini Palazzoli highlighted cultural change as a driving force behind the occurrence of EDs. Palazzoli noted that the earliest clinically accurate descriptions of AN (see section on EDs throughout history below) occurred in London and Paris in the early 1870s, “two cities in the grip of a cultural crisis” (Palazzoli 1974:245) and argued that in a contemporary parallel, the agricultural-patriarchal family system was undergoing equally fundamental changes: “[T]hroughout history the family has always served as a homeostatic barrier against social change, and never before has it had to face more rapid and radical changes than today. Hence the universal feeling of malaise with which modern family life is suffused.” (Palazzoli 1974:248)

For Palazzoli, this situation called for a larger involvement of the patients’ families in treatment and therapy. This idea is still predominant, at least in the treatment of children and adolescents; in fact, family-based treatment is still one of the few treatment measures for EDs that have substantial evidence in their favor (Gowers et al. 2007; Lock et al. 2010). However, whereas Palazzoli was very blunt in condemning what she saw as cold, critical and passive-aggressive traits in the parents, modern family-based treatment stresses that any specific causes behind the illness are usually not easily discoverable and that an explicit de-emphasis of causality can strengthen caregivers in focusing on the ‘here and now’ of treatment, instead of being paralyzed by endless rumination over how they have gone wrong as parents (Lock and Le Grange 2013). Girard, while clearly implicating changes in family dynamics as a causative factor, appears to side with the latter notion: “Anorexia is a phenomenon that appears in an era in which the family is breaking down. To try at all costs to find an explanation for it in the patients’ families is to stay locked in a schema that is less and less relevant.” (Girard et al. 2013:68) In other words, individual families are not to blame—it is not their fault that the family as sociocultural entity is losing its authority as safeguard against mimetic rivalry.

The scope of this article does not allow for a more all-encompassing review of causality in EDs. Usually, like in other areas of psychiatry, a biopsychosocial model is applied (Culbert, Racine, and Klump 2015; Strober and Johnson 2012). Nevertheless, in order to investigate Girard’s emphasis on cultural change, it is worth broadening the scope somewhat to review the evidence on cultural change in the form of migration and acculturation as a causal factor. The question of ‘Westernization’, industrialization, urbanization, etc. as driving forces in the changing patterns of ED prevalence on a global scale has been the subject of much debate (see for example Nasser, Katzman, and Gordon 2001; Lester 2004) and it has been suggested that AN could be construed as a Western culture-bound syndrome (Prince 1985). A recent review paints a complex picture: the majority of evidence suggests that there is an association between such cultural change and ED psychopathology, but both greater and lesser levels of acculturation have been identified as potential risk factors and ethnicity may serve as a moderating factor (Doris et al. 2015). Interestingly, in a review of ED rates over time and of cross-historical and cross-cultural evidence, the authors concluded that BN is a cultural-bound syndrome while AN is not (Keel and Klump 2003). Likewise, urbanization has been implicated as a risk factor for BN but not for AN (Hoek et al. 1995). In a review of the emergence of EDs throughout the Asian continent, the authors conclude that EDs “are not culture-specific or culture bound, but perhaps better thought of as culture-reactive, whereby certain cultural contexts result in social and environmental changes that increase risk for EDs” (Pike and Dunne 2015:9). Here, different societies respond differently. Taken together, there appears to be support for viewing cultural change as a driving factor in the development of EDs, but the causality is most likely complex, multidimensional, and unstable, “[i]n contrast to theoretical models positing uni-dimensional, orthogonal, or oblique relations between cultural identities undergoing acculturation” (Becker et al. 2010a:755).

As seen, the view that mimetic forces originating in an archaic dawn of mankind have formed an ever-present, yet virtually invisible societal fabric that has eventually been torn apart by modernity is central to Girard’s worldview in general as well as to his ideas about the surge of EDs. In the next section, this fateful historicity is explored even further.

## Eating Disorders In and Out of History

There is a substantial literature on the phenomenon of *anorexia mirabilis*, or ‘miraculous lack of appetite’, describing women and girls in the Middle Ages and onwards who starved themselves in the name of God. A portal figure in this exposition is Catherine of Siena, a 14th century Dominican tertiary whose habits of ‘holy fasting’ is recurrently highlighted as an early description of AN, implying a historical continuity between these conditions (Bell 1987; Vandereycken and van Deth 1994). Others have argued that such continuity may at least in part be illusory, stemming from an anachronistic application of the modern disease paradigm in a historical context where extreme fasting was regarded as a legitimate form of religious piety (Bynum 1987; Brumberg 1988). It has later been suggested that this discord is due to a one-sided emphasis on either individual psychopathology or cultural context and that more complex, synergistic models can be imagined (Lester 1995). Later cases of anorexia mirabilis during the Victorian era appears to have had more ambiguous motives, usually emanating from piety but not seldom developing into early equivalents of tourist attractions, drawing audiences from across the European continent and thus entering a reinforcing circle of curiosity and deviance (Keel and Klump 2003; Russell and Treasure 1989).

While acknowledging the potentially mimetic competitiveness of religious fasting, Girard contends that the medieval convents knew how to handle this minor nuisance, affecting—according to Girard—a few hundred people at most compared to the millions worldwide suffering from EDs today (Wade, Keski-Rahkonen, and Hudson 2011):

The strange thing is that medieval convents were much more aware of the danger than we are in the modern world. Handbooks of asceticism took it into account. In the Middle Ages, there was competitive fasting among persons who wanted to earn a reputation as ascetics. There was a positive goal, a veritable ambition to attain dominance, analogous but not identical to modern anorexia, which is linked to the gaze, to the universe of photography. (Girard, Anspach, and Tacou 2013:58–59)

Although she is not mentioned in Girard’s writing, this passage again echoes Palazzoli:

This type of asceticism [i.e., AN] should not be confused with the religious: the saint becomes an ascetic not as an end in itself but as a means to attain mystical communion with God and all His creatures. […] Great religious teachers have always been alert to the psychological, no less than to the physical, dangers of penitential fast, and they have warned particularly against the mistaken sense of power and euphoria that often affects those who engage in such practices. (Palazzoli 1974:74–75)

However, unlike Palazzoli, Girard repudiates the common notion that most societies throughout history have favored a rotund body ideal associated with a well-fed elite. He criticizes what he sees as an overall tendency of wrongfully “attributing to the entire European past an inordinate predilection for fat women, rooted, we claim, in an obsession with food resulting from the state of semi-starvation which was normal in those days” (p. 14). Girard argues that in preindustrial Europe, a large majority of people lived on farms and it would therefore have been unthinkable even for the most tyrannical rulers to starve their own people, thereby risking their own supply of food. Anyhow, the occurrence of food shortages would have been unlikely to affect contemporary aesthetic ideals:

[E]ven if food shortages had been as common as now claimed, it is most doubtful that they would have influenced the conception of feminine beauty held by painters and sculptors. In these days, aesthetic fashions did not originate with the lower classes but with people too closely associated to the ruling circles not to share in their privileges, at least as far as food was concerned. Even in times of famine, artists were certainly among the last to go hungry. There is nothing to suggest that they dreamed about food half as much as we do. The fatness imperative we clamp upon the past is a crude projection of our own obsession with food, an obvious maneuver to deny our own singularity. (pp. 14–15)

Although British physician Richard Morton is often credited with having made the first clinical description of AN in 1689—referring to it as “a Nervous Consumption” (Silverman 1983:2830)—two later figures are usually considered to be the original ‘discoverers’ of the disorder, independently providing almost simultaneous clinical descriptions in the early 1870s: British physician William Gull (Gull 1874) and French physician Charles Lasègue (Lasègue 1873), whose work are often used as historical reference points (Russell and Treasure 1989). Modern commentators have also argued that the somewhat mysterious and now extinct disease known as *chlorosis*, or ‘green-sickness’, was not simply a form of anemia but a functional disorder related to AN (Loudon 1980). On his part, Girard is very precise regarding the emergence of EDs: “It all began, as it should, as in a fairy tale, with some beautiful and prestigious women in very high places. The most important of these mimetic models was Elizabeth of Austria [1837–1898], the wife of Emperor Franz Joseph […].” (p. 12) Girard argues that Empress Elizabeth kept a rigidly restrictive diet and engaged in excessive physical activity, setting in motion a mimetic spiral among the noblesse:

Together with the wife of Napoleon III, Empress Eugenie of France, another famous beauty, [Elizabeth] put an end to the crinoline that imprisoned the lower part of a woman’s body. At some encounter of their two imperial husbands, these great ladies retired to a private room for the purpose, we are told, of comparing their respective waistlines. This incident suggests some kind of incipient competition between the two, exactly what was needed to start a pattern of mimetic rivalry among the numerous aristocratic ladies who had nothing to do but to look up to [Elizabeth] and Eugenie and copy their behavior down to the last detail. The two empresses certainly played a role in the triggering of the mimetic rivalry that has been widening and intensifying ever since. After World War I, the escalation reached the middle class and after World War II, at least in the opulent West, it spread to all social classes. (p. 12)

An important watershed in the debate on the historicity of EDs is the question of what constitutes the central psychopathology in AN. If weight phobia is considered a necessary component in distinguishing AN from other causes of undernutrition, the medieval cases of extreme religious fasting or the cases of anorexia mirabilis of the Victorian era cannot be viewed as EDs in the modern sense even though they clearly describe young women engaging in harmful self-starvation (Keel and Klump 2003). Weight phobia has been described as an emerging aspect of AN during the 20th century (Russell and Treasure 1989) and was not seen as a vital part of the conditions originally described by Gull and Lasègue (Keel and Klump 2003). Notably, this has also been the case in ED research in Chinese patients in Hong Kong, where *non-fat phobic* AN was described in the 1990s (Lee, Ho, and Hsu 1993), only to be gradually superseded by a more ‘orthodox’ clinical picture of weight phobia and distorted body image (Pike and Dunne 2015).

Although BN was not described as a separate entity until the late 1970s, several attempts have been made to trace historical cases (Keel and Klump 2003). For example, Roman emperors Claudius and Vitellius have been portrayed as possibly being afflicted with BN (Crichton 1996), but analogous to the cases of anorexia mirabilis discussed above, the imperial episodes of overeating and vomiting of the 1st century A.D. appears to have been culturally sanctioned and stemmed from sovereign extravagance rather than from negative body image (Ziolko 1996). Girard is clear in his opinion: “[T]he decadent Romans were innocent sensualists. They, too, were eating and vomiting in turn, but for themselves only and not for anybody else. […] Our modern bulimic is eating for herself, to be sure, but she is vomiting for others, for all these women who are watching each other’s waistlines.” (p. 8)

The debate around ‘holy fasting’ as a historical version of AN flared up in the 1980s and 1990s but seems to have subsided somewhat during the past decade without any consensus being reached. In the clinical ED field, it has nevertheless become an oft-repeated and almost canonical truth that AN as a clinical entity can be traced to ancient times (see for example Espi Forcen 2013; Harris 2014). However, a unifying view can be imagined and is gaining support in genetics research: that the ability to maintain a low weight is an innate trait that renders certain persons more vulnerable to detrimental weight-loss (Nilsson et al. 2013), be it in the context of piety or as part of an ED. Most people that try and succeed in losing weight do not manage to keep this new desired figure for a sustained period of time—dieting becomes tedious and boring, initial dedication fades, old habits reestablish themselves, etc. The fact that body weight is usually maintained at a relatively stable level over time in spite of large fluctuations in energy intake implies the existence of a biological ‘set-point’ in human weight regulation (Farias, Cuevas, and Rodriguez 2011). In a minority of individuals, however, weight loss may trigger further weight loss in a dangerous spiral, due at least in parts to a trait-like ability to maintain a low body weight in spite of undernourishment. Such a trait may at times throughout human evolution have been advantageous, conserving it until modern times (Bulik and Allison 2002). Moreover, traits of perfectionism have been associated with asceticism in AN but would also have facilitated devotional fasting, although the motives were different (Fassino et al. 2006).

## Conspicuous Non-Consumption

The recurring references to blood feuds and ritual sacrifice may also give the impression that Girard’s ideas are more readily applicable on pre-modern societies. Indeed, in Girard’s account, the fabric of mimesis bestows everyday life with an archaic quality. However, Girard argues that the rise of acquisitive individualism and upward mobility in the Era of Industrialization actually led to accentuated mimetic rivalry. Here, he invokes the theories of sociologist Thorstein Veblen, who in the 1920s introduced the concept of *conspicuous consumption* to explain the emergence of a tendency to uphold social status by display of consumption patterns, leisure time, etc. (Veblen 2009; see also Corrigan 1997). It is in this context that Girard situates the emergence of AN, describing it as a form of “conspicuous non-consumption” comparable to how well-off people sometimes dress down in order to display “a calculated indifference to clothes, an ostentatious rejection of ostentation” (p. 10). He writes:

When the wealthy become accustomed to their own wealth, straight conspicuous consumption loses its appeal and the nouveaux riches turn into anciens riches. They perceive this change as the summum of cultural refinement and they do their best to make it as conspicuous as the former consumption. They invent a conspicuous non-consumption, therefore, superficially discontinuous with the attitude it supersedes but, at a deeper level, it is a mimetic escalation of the same process. (p. 10)

In the passage quoted above, one may get the impression that this is a conscious process. Girard, however, is ambiguous on this point, also pointing out the self-reinforcing nature: “Often, fashions have no meaning; people simply imitate them without reflecting on their significance. The individual becomes a vehicle for a significance that eludes him.” (Girard et al. 2013:63) Mimesis, as noted in the introduction, becomes an automatic process that may occasionally appear in paradoxical forms. Girard compares the non-conspicuous mimetic aspects of EDs to the anthropological phenomenon of *potlatch*—the circulation of sumptuous gifts that forms the basis of the economic systems of the indigenous people of the Pacific Northwest Coast (see for example Jonaitis 1991; Mauss 1966):

The normal purpose of exchanging gifts, in all societies, is to prevent mimetic rivalries from getting out of hand. The spirit of rivalry is so powerful, however, that it can transform from the inside even institutions that exist only for the purpose of preventing it. The potlatch testifies to the formidable stubbornness of mimetic rivalry. It may be defined as a frozen slice of mimetic crisis that becomes ritualized and finally plays a role, but at great cost, in the control and attenuation of the competitive fever. In any society, competition can assume paradoxical forms because it can contaminate the activities most alien to it in principle, especially the gift. (p. 11)

For Girard, potlatch testifies to the fact that “[t]here can be rivalries of renunciation rather than acquisition, of deprivation rather than of enjoyment” (p. 11). Interestingly, in his book *Things Hidden Since the Foundation of the World*, originally published in 1978—almost two decades before engaging in the debate around EDs—Girard acknowledged the possibility of an inverted variant of mimesis, as seen in the potlatch, “in which acquisitive mimesis is inverted into a mimesis of renunciation and is capable, like its opposite, of attaining a disastrous intensity” (Girard 2016:9). In *Eating Disorders and Mimetic Desire*, he discovered this mimesis of renunciation on the body of the AN patient. This idea of the body as an arena for the display of “concretised metaphors” is certainly not new to the field of ED research (Skårderud 2007:246). However, it has been noted that bodily metaphors tend to implode in patients suffering from AN, whereby “the ‘as-if’ of the metaphor as a figure is turned into an ‘is’”, the metaphor thus becoming “too real” to be of any use (Skårderud and Fonagy 2012:353). Thus, the patient with AN *is* his or her weight. Girard makes the exact same observation:

[T]he metaphor is turning into a massive existential fact, resulting in an uncanny and enlightening reversal of the conventional relationship between metaphor and reality. When our relativists maintain that only metaphors exist, they do not realize how right they are. They underestimate the power of certain metaphors to become terrifyingly real. (p. 19)

As violence is directed toward the self, the mimetic theory appears to become inverted. Instead of a vicious circle of increasing abstraction and uncoupling, as in the case of the blood feud, undernourishment unconditionally leads to spiraling levels of concreteness. The Minnesota Starvation Experiment of the 1940s, in which 36 conscientious healthy young men were subjected to a semi-starvation diet of turnips and dark bread for six months, showed that the same obsessiveness arises regardless of the reason behind food restriction (Keys 1950; Kalm and Semb 2005). For any starving person, everything revolves around eating—hence the high anxiety levels in patients suffering from AN, forced by their undernourished brains to think about frightful food non-stop. For Girard, “the drive toward less and less can substitute for the drive toward more and more and ultimately mean the same thing” (p. 11). The autonomy and self-sufficiency signaled by abstaining from something actually qualifies as an immensely powerful generator of mimetic rivalry: “Ultimately this process may turn into a complete rejection of competition, which is not always but may be the most intense competition of all.” (pp. 10–11)

There is ample evidence of mimetic forces at play in the emergence of EDs. In 1954, social psychologist Leon Festinger proposed a *social comparison theory*, describing how individuals compare themselves to others in order to self-evaluate (Festinger 1954). Upward social comparison—or mimesis if you will—is an important feature of this concept. However, contrary to Festinger’s original suggestion that comparisons mostly occur among people similar to ourselves, it has been shown that women frequently compare themselves to unrealistic and elusive media images and continues to do so even in the face of adverse health impact (Strahan et al. 2006; Leahey, Crowther, and Mickelson 2007). Undoubtedly, an increasingly slim body ideal evolved in the second half of the 20th century. An analysis of storefront mannequins shows that while their body proportions were mostly in the normal range before the 1950s, since at least the 1990s they differ from those of normal young women to the point that in reality they would probably not be able to menstruate due to low amounts of body fat (Rintala and Mustajoki 1992). Studies by anthropologist and psychiatrist Anne Becker have shown that disordered eating became significantly more prevalent after the introduction of television to a media-naïve population in Fiji during the 1990s (Becker et al. 2002). A similar association was subsequently found upon social media exposure (Becker et al. 2011; see below for a more detailed account of Becker’s research). Peer modeling and endorsement have been shown to be some of the most powerful causes of dieting behaviors in adolescents (Allison et al. 2014; Quiles Marcos et al. 2013). Although current sociocultural models often fail to explain the exact mechanisms behind how thin-ideal internalization can induce disordered eating, upward social comparison has been isolated as such a mediator (Fitzsimmons-Craft et al. 2014). These mimetic properties are perceived at an early age. In a study in which preschool children were interviewed about pictures of kids with different body types, the chubby kid was rated most negatively and was less often picked as a potential playmate (Musher-Eizenman et al. 2004).

But if mimetic desire forms such an integral part of our contemporary society, how come not all of us fall victim to it? Certainly, being immersed in an ever-present slenderness imperative is not enough to develop an ED. While large proportions of the population experience body dissatisfaction, the prevalence of AN among young women is only approximately 0.4% (American Psychiatric Association 2013). Girard explicitly acknowledges this fact:

We all compare ourselves to others, we are all prone to mimetic rivalry, but not everyone carries this tendency to the point of pathology. Why does anorexia strike some women more than the rest? Individuals are more or less rivalrous; this is just as true where thinness is concerned as in other areas. (Girard, Anspach, and Tacou 2013:66–67)

This is not an uncommon theme in the treatment of EDs. Most people create and evaluate a self-image based on their performance in a variety of domains in everyday life: relationships, school, work, sports and hobbies, parenting, and—yes—weight and body shape, etc. The self-image of a person with an ED is not categorically distinct in this regard; the major difference is not that they compare themselves to others, but that they judge themselves solely on their ability to control their eating habits, weight and shape. Illustrating this in the form a pie chart, where the subjective importance of different parts of an individual’s life are estimated, can be a powerful tool in making a person with an ED aware of this cognitive bias (see for example Fairburn 2008). Furthermore, persons with an ED have also been found to experience high levels of shame in contexts of social comparison, to be highly self-critical, to resort to their ED symptoms in developing a sense of personal pride, and to have a low capacity for being open to using compassion from self and others as a soothing mechanism (Goss and Allan 2014). From a Girardian point of view, these traits all risk aggravating the influence of mimetic rivalry to a point where the individual loses control of the situation and pathology develops.

There is, however, within the field of ethnographic ED research a contrasting view, framing EDs primarily as ways of engaging in subjective self-care, albeit in skewed and desperate forms, rather than as mimetic competitiveness influenced by societal slenderness imperatives. It is well established that ED behaviors are not only associated with negative affective states, but that they may also bring about positive feelings of pride, accomplishment and will-power, at least in the initial phase of the illness process (Selby et al. 2015). Anthropologists Megan Warin and Connie Musolino have shown how individuals with long-standing restrictive EDs can depict their ED behaviors as morally virtuous forms of caring for themselves, providing safe spaces within a framework of culturally accepted *healthism* where constant dieting and physical exercising are promoted as parts of a healthy and desirable lifestyle (Musolino et al. 2015a; Musolino et al. 2016). This may also provide clues as to why many persons with EDs are reluctant to seek medical help: they do not view their eating habits primarily as problematic but as taking *extremely* good care of themselves. Anthropologist Anna Lavis has also written extensively about this tendency:

In clinical terms, the self-starvation of anorexia nervosa is regarded as lacking in self-care and propelled by a loss of agency to the illness. This conceptualizes food not only as *the* vector through which to care for an individual with anorexia, but also as that which (re)produces their self-care, where that comes to mean, literally, care of the self. […] In the clinic, food is thus ’an essential therapeutic intervention’ not only for the integrity of the suffering body but also for the recovery of selfhood. However, engaging with individuals with anorexia elucidates not only how such care may be experienced as care-less, but also that embodied practices of not eating engender alternative models of attention; anorexia, although recognized by participants as an illness, offers ways of caring both for oneself and for Others. (Lavis 2015:81–82)

A database study on self-image and treatment outcome in patients with AN and BN supports this view, as higher scores on subscales measuring subjective wish to protect and preserve the self indicated a poorer prognosis (Birgegård et al. 2009). If such self-care is actually seen as synonymous with continued ED behaviors, this is not surprising. Of course, this framing is not completely incompatible with the concept of mimetic desire, but where Girard’s model inherently focuses on the conspicuous violence of disordered eating, these researchers point instead to the opposite perspective of subjective self-care. Indeed, both Lavis and Warin invoke aspects of belonging and relatedness as central in maintaining an ED. Lavis notes that “[a]lthough anorexia as skin and space seems de-relational, there is a complex sociality to many [patients’] narratives” (Lavis 2015:94), an ambiguous and largely contradictory relatedness that Warin describes as at once competitive and soothing, “as if belonging to a secret and powerful group—‘a religion’, ‘a competitive sporting team’, or part of ‘a game’” (Warin 2006:43). In her book *Abject Relations: Everyday Worlds of Anorexia*, Warin further describes this equivocal nature: “[Informants] simultaneously experienced pleasure and disgust, were empowered and disempowered, felt safe yet constantly threatened, were both pure and dirty, and when sickest felt at their best. Anorexia was a constant process of becoming and unbecoming, of having a life by moving toward death.” (Warin 2010:4) Based on observations such as these, Lavis has explicitly criticized Girard’s emphasis on mimetic desire for thinness as central to AN, rejecting it as superficial privileging of the dreadfully captivating visual aspects of the disorder which “frames anorexia as a means to a desired end [i.e., thinness] rather than the object of desire [i.e., an ambiguous sense of belonging] itself” (Lavis 2016a:70). Such a tendency—objectionable or not—is, one might argue, certainly not an invention of Girard’s, but it is central to his understanding of EDs. Again, alternate models of desire may very well be compatible with Girard’s concept of mimesis and could potentially deepen his analysis, but are unfortunately not further explored in his account.

There is also an ethnographic critique that focuses on how gendering of mimetic forces has created a “perceived confluence between eating disorders […], mediated images and highly ‘susceptible’ female audiences” (Holmes 2016:1). Sociologist Abigail Bray argues that EDs are commonly framed as something akin to *reading disorders*, whereby young women’s alleged inability of applying a critical approach in their consumption of media images and resisting destructive influences is seen as being at the root of the problem. Bray writes:

The idea that representations are harmful to women’s bodies became popular during the nineteenth century, when biomedical professionals entered into the debate over women’s education. Hysteria and neurasthenia were framed as the ‘new woman’s’ diseases: the more educated the woman, the more likely she was to suffer from nervous complaints. […] The consumption of popular literature and magazines was also linked to the enfeeblement of the mind and body. […] In other words, the infantilization of women’s reading practices as necessarily detrimental to their minds has an extensive history within modernity. (Bray 1996:418)

In contrast to the findings from Becker’s research on media-naïve Fijian adolescents, Bray argues that television in particular has come to unfairly represent the contagiousness of EDs; for Bray, “to argue that women contract psychiatric diseases from television is to enter the realm of science fiction” (Bray 1996:419). Of course, Becker does not suggest that EDs attack unsuspecting youth through the plasma screen; even so, Bray’s comment illustrates a valid critique of the mechanisms of mimesis. In a similar vein, the theory of conspicuous consumption that Girard relies heavily on has been critiqued for being too simplistic in reducing consumerism and fashion to largely irrational female domains, lacking in agency and autonomy (see for example Wilson 2003).

Whereas Girard does not necessarily blame uncritical media consumption for the rise of EDs, his account of the mimetic properties of what he describes as a societal slenderness imperative appears to suffer from a similar gender bias. In the opening paragraph of *Eating Disorders and Mimetic Desire*, Girard acknowledges that both women and men are afflicted by EDs; however, in the ensuing account he consistently writes about women. This gendering is not apparent in his broader work on mimetic phenomena and may simply be a result of the popular stereotypical labeling of EDs—AN in particular—as feminine (see for example Boysen et al. 2014). In some passages, however, it does appear as though Girard is inscribing himself into a tradition of reducing AN to “a carnivalesque image that is represented by femaleness, thinness, illness, horror, fascination, and death” (Warin 2004:96). Although a thorough review of the large literature on feminist conceptualizations of EDs (see Bordo 1993; Lester 1997; Musolino et al. 2015b; Orbach 1986) is beyond the scope of the present article, it is worth mentioning that *desire for sameness*—which is, as we have seen, a condensed phrasing of Girard’s model of mimetic desire—has repeatedly been described in feminist literature as a perceived female threat to male structures of normality, thus being rendered as unhealthy and contagious (Burke 2006; Irigiray 1985). Of course, popular labeling notwithstanding, many men are afflicted by an ED and there appears to have been a parallel mimetic tide of unrealistic and unattainable male body ideals in the past decades, albeit with a focus on muscularity rather than slenderness (Pope, Phillips, and Olivardia 2002).

## A Few Missing Pieces

As we have seen, Girard argues that in contrast to contemporary models, EDs are best explained in terms of ever-present mimetic forces readily recognizable by each and everyone of us. This, however, raises questions about whether there are in fact societies and contexts that cannot per default be included in his mimetic community and to which his arguments are not as easily transferable. In the first pages of *Eating Disorders and Mimetic Desire*, Girard rejects Freudian concepts by relating an episode from his youth:

Just before World War II, a pretty cousin of mine was dieting furiously and her father, my uncle, was storming about helplessly, trying to get her to eat more. Fathers, as a rule, are not pleased to see their daughters starve themselves. This particular father was also a physician, at a time when the medical profession had not yet caught the disease it was already trying to cure. This uncle was our family doctor and, as such, had great prestige in my eyes, at least until that day. I had not yet read Freud but my later skepticism regarding his conception of fatherhood may well originate in this incident. I immediately perceived that my cousin was listening to a command more powerful than her father’s desire and, with the passing of time, this more authoritative voice has become louder and louder. It emanates from the people who really count in our adolescence and who are our peers and contemporaries rather than our fathers. The individual models of young people reinforce the authority of the collective models which are the media, Hollywood, and television. The message is always the same: we have to get thinner, regardless of the cost. (pp. 2–3)

Here, a number of telling observations can be made. First, the main protagonist is firmly established: a young French upper middle-class girl who diets in order to get thin. This is, of course, the predominant stereotype of the ED patient and not an invention of Girard’s, but it is very likely that this early experience shaped his overall understanding of EDs.

Secondly, we need to ask if the message really is, universally, that we have to get thinner. This does not appear to be the case. A number of ethnographic studies have highlighted cultural contexts where fatness, rather than slenderness, is associated with attractiveness, marriageability and fertility as well as with attributes such as generosity, religiosity and social prominence. Anthropologist Rebecca Popenoe has shown how for seminomadic Azawagh Arabs in Niger, fattening practices among women have the power to transform accumulated wealth into honor, sexiness and fertility (Popenoe 2004). In her studies among Puerto Ricans in Philadelphia, anthropologist Emily Bradley Massara found positive associations between fatness and marriage (Massara 1989). Similarly, whereas anthropologist Mimi Nichter found high levels of stereotypical weight talk among White girls in her studies of American middle and high school students, African-American girls seemed to largely escape this. For them, beauty had more to do with projecting attitude and moving with confidence than achieving slenderness and they were generally much more satisfied with their bodies than were the White girls (Nichter 2001). Anthropologist Elisa Sobo has described how in rural Jamaica, fat bodies represent beauty and vitality based on agricultural analogies whereby fatness connotes ripeness and juiciness (Sobo 1994); however, there is an ambiguity in these connotations, as ultimate ripeness is inevitably followed by decay. This is also the case in Jessica Hardin’s study of fat talk in Samoan Christian communities, where fatness is seen as a positive symbol of strength and prestige but, as so often happens with such markers of high status, it may also become the target of subversive comments implicating greed and corruptness (Hardin 2015).

In discussing studies such as these, there appears to be ripe opportunities to apply a Girardian mimetic perspective—if, however, a slightly different one than what is outlined in *Eating Disorders and Mimetic Desire*. Unfortunately, this is, somewhat prematurely, discarded in Girard’s writing. As we have seen, he does acknowledge that “obesity is even more on the rise than extreme slenderness”, but in his account, overweight persons are described as “drop-outs” (p. 17), exhausted by a universal race for thinness and serving merely as an exception that proves the rule. The outright Girardian rejection of the historical fatness-as-beauty trope has already been discussed above. This rejection is, however, based on the assumption that fat bodies signaled wealth in times of semi-starvation—something that Girard does not believe in—but, as seen in Sobo’s research in Jamaica, positive connotations around fatness could also very well arise under flourishing agrarian circumstances. Undeniably, this is a blind spot in Girard’s writing. A sharply different account is, for example, offered by fellow literary critic Mikhail Bakhtin in his seminal work on the medieval world of François Rabelais (Bakhtin 1984). In Bakhtin’s writing, carnivalesque depictions of food and fatness in the European Middle Ages do not primarily occur in the realm of the ruling classes against a backdrop of perpetual starvation but as active and triumphant means of moving towards a better, more just future for the common people and society as a whole. In Bakhtin’s words:

The banquet takes place, as it were, in a new epoch. And one might say that the carnival banquet was also held in the utopian future, in the Saturnian age come back to earth. [… B]anquet images in the popular-festive tradition (and in Rabelais) differ sharply from the images of private eating or private gluttony and drunkenness in early bourgeois literature. The latter express the contentment and satiety of the selfish individual, his personal enjoyment, and not the triumph of the people as a whole. (Bakhtin 1984:301–302)

Here, the utopian view of Bakhtin stands in sharp contrast to Girard’s downplaying of the very concept of body ideals throughout history. However, while fatness is certainly not universally frowned upon, there are studies that show that slender body ideals are spreading globally. Medical anthropologist Alexandra Brewis and colleagues have shown that in parallel with a global rise of overweight and obesity rates in all regions except in sub-Saharan Africa, a somewhat contradictory trend of both slim idealism and fat negativism has emerged in places where fatness has traditionally not been looked down upon (Brewis et al. 2011; see also Brewis and Wutich 2012). In this particular study, the highest fat stigma scores were not found in the United States or Europe, but in Paraguay, Mexico, and Samoa, although the authors hypothesize that this is in part due to a larger portion of politically correct self-censoring in the Western study sites. Increasing levels of weight-related stigma—which often includes moral judgements such as being lazy, irresponsible, ugly, and lacking self-control—has recently been described in Guatemala (Hackman, Maupin, and Brewis 2016) and South Korea (Brewis, Han, and SturtzSreetharan 2017), where it has been reported that the level of disturbed eating and body dissatisfaction is one of the highest in the world (Pike and Dunne 2015).

Recent research also nuances the picture provided by Nichter and Massara, as discussed above. Anthropologist Stephanie McClure has found that in a group of African-American high-school girls in the United States Midwest region, while the levels of body satisfaction and acceptance of large body size were comparatively high, there was also a large variety in how the girls experienced their weight and body shape (McClure 2013). Thus, McClure notes that “the body conceptualization and practices attributed to African American females as a function of their racial affiliation, while not untrue, are incomplete—that there is no ‘The African American Female’” (p. 304). Notably, in a study of barriers to receiving ED treatment, African-American informants reported that stereotypical assumptions among health care staff about who can and cannot suffer from an ED had resulted in their ED symptoms being neglected and their treatment delayed (Becker et al. 2010b). One informant described how such assumptions had become internalized, so that she felt that as an African-American woman she *should* simply not have an ED: “not only is your health compromised, but your identity is compromised too” (p. 637). Another recurrent barrier was that health care staff tended to equate EDs with emaciation, making it especially difficult for obese persons to get proper treatment.

Interestingly, there are several mimetic aspects related to fatness and fat stigma. People with higher or lower body-mass index tend to clump together socially over time, partly due to shared social behaviors surrounding exercise etc. (Hruschka et al. 2011). Non-overweight adolescents are more likely to select equally non-overweight friends, leaving overweight adolescents with few other choices than to turn to each other for friendship (Schaefer and Simpkins 2014). This can, in turn, perpetuate low levels of physical activity among obese individuals (Simpkins et al. 2013) and lead to higher risk of depression, as seen among those with more constricted social networks (de Wit et al. 2010). These mimetic mechanisms were, however, not apparent to Girard, who merely notes that “poor women are fatter than others because they eat fattening food, and also because they do not stint themselves” (Girard et al. 2013:55). While such a statement may technically be valid, it lacks any explanatory finesse and appears as rather nonchalant alongside his usual style of argumentation.

A few case studies may highlight how the topic of slenderness and fatness requires a more nuanced approach. For example, there are modern societies where EDs are almost unheard of even though mimetic processes involving beauty and body shape are ever-present. This is illustrated by the work of medical and psychological anthropologist Eileen Anderson-Fye, who spent several years doing fieldwork among high-school girls in San Andrés in Belize. Here, beauty pageants are ubiquitous and Anderson-Fye reported how young women constantly engaged in talk about whether classmates or celebrities had a ‘Coca-Cola’ or ‘Fanta’ shape; i.e., an ideal hourglass shape or a more straight shape (Anderson-Fye 2004). Still, informants were generally content with their own body shape and size, and even though they clearly recognized ‘beauty’ as a nationally cherished characteristic, they valued qualities such as being a kind and friendly person more. Apparently, mimesis does not necessarily entail rivalry. Similarly, a separate study on eating attitudes and behaviors revealed that although the Belizean girls’ beliefs about eating and body shape overlapped somewhat with Western ideals, they displayed low levels of actual pathological eating behaviors (Anderson-Fye and Lin 2009). Interestingly, Anderson-Fye describes how a culture-specific practice of self-protection, known to the Belizean girls as ‘Never Leave Yourself’, mediates how they make sense of and deal with experiences and practicalities of everyday life. “As this implicit concept is passed and lived orally”, Anderson-Fye notes, “most girls had not written down the signifying phrase until I asked them to spell it” (Anderson-Fye 2003:66); yet, this idea of staying true to one’s own ideals and feelings in the face of obstacles or temptations was ever-present in the teenaged informants’ stories of how they navigated their society. ‘Leaving oneself’ could occur if, for example, a girl did not pay respect to her cultural ancestry, did not tell the truth even if it meant upsetting another person, or did not stand up for herself and speak back to someone who had wrongly accussed her. Here, the ‘Self’ would take center stage even in situations of obvious peer pressure:

[T]his generation of San Andrés adolescent schoolgirls seems to be resolving the fundamental paradox in the opposite direction as the U.S. schoolgirls. That is, the majority of the San Andrés girls were willing to give up any “Relationship”—friends, boyfriends, parents—if it required them leaving themselves. […] This is not to say that girls did not expend heroic effort trying to imagine, secure, maintain, and repair relationships, even after horrific betrayals. In contrast, the Belizean girls put great effort into their relationships, but with a few exceptions among the schoolgirls they did not often do so at the expense of their well-being […] or their health. (Anderson-Fye 2003:72)

This also meant that in spite of the blatant body talk and apparent obsession with Western mass media among the girls, their ‘Never Leave Yourself’ attitude appeared to spare them from body shame, dieting and excessive exercise, since these notions run counter to the self-care ethos of the Belizean society. However, Anderson-Fye also notes that those girls that actually did display disturbed eating behaviors often had ties to the growing tourism industry and that the Belizean cultural emphasis on self-care may slowly be diluting.

Anne Becker’s research in Fiji have briefly been touched upon above. In her original ethnographical studies carried out in the 1980s and 1990s (Becker 1995), Becker describes a Fijian society where there is, as in Anderson-Fye’s Belize, indeed a consensually admired body ideal: largeness is highly valued and there is an explicit attentiveness to the body shape and weight of others. So far, so good for the Girardian take on mimetic rivalry—but what Becker then goes on to describe is a culture where the preferred body type has little to do with personal strivings or shortcomings and everything to do with social connectedness and the sense of being cared for:

However, while there seems to be a consensual preference for particular ideal physical attributes in Fiji, there is a striking absence of interest in attaining these as a personal goal. Moreover, given the close attention directed toward others’ body shapes and sizes reflected in constant commentary, there is a paradoxical denial of interest in cultivating one’s own shape to appoximate the ideal. (Becker 1995:38)

Here, since the shape of one’s body is not dependent on personal ambition and self-control but on the communal matrix that one is embedded in, individual attempts to alter size and weight do not make any sense. Again, the occurrence of mimetic structures does not automatically entail rivalry on an instrumental level. In fact, in Becker’s research, largeness had very high positive connotations of being cared for among the Fijians *despite* the fact that her study population gave relatively low attractiveness ratings to overweight and obese figures. As Becker notes, the Fijian body “is the responsibility of the micro-community that feeds and cares for it; consequently, crafting its form is the province of the community rather than of the self” (Becker 1995:57).

Another noteworthy example is the research by anthropologist Emily Yates-Doerr on practices of care associated with food and eating among obesity patients at a Guatemalan public hospital. Yates-Doerr describes how a communal logic of care, similar to that of the Fijians, interfered with a conventional person-centered nutritionist approach and made such advice if not redundant then at least largely out of touch with the everyday lives of the patients: “Despite the message of individual choice implicit in nutrition protocol, the nutritionists found that an emphasis on personal food choice produced the feeling of anxiety similar to that found in restrictive eating. They viewed eating practices as social, and because of this, as practices that could not be managed by willpower alone.” (Yates-Doerr 2012:151) When Yates-Doerr visited the home of one of the patients from the clinic, she found that the woman owned a single-burner stove and one single cooking pan—this patient could not possibly follow the nutritionist advice to prepare separate meals for herself and for her family (Yates-Doerr and Carney 2016). This illustrates how mimetic mechanisms are often, if not completely absent or void, intimately bound to and largely trumped by socioeconomic boundaries, material resources, community matrices, etc. In contrast, in *Eating Disorders and Mimetic Desire*, the processes of actually acquiring food, preparing and cooking it are hardly mentioned at all.

## Conclusion

This account has touched upon three main themes in Girard’s thinking: the ‘end of prohibitions’ as a driving force in the emergence of EDs, EDs as a phenomenon specific to modernity, and the significance of conspicuous non-consumption. As shown, these ideas are certainly not unique to René Girard; however, to the best of my knowledge, Girard was the first to attempt to link them in order to create a cohesive account of the rise of EDs in the modern era. Undoubtedly, the debates he engages in *Eating Disorders and Mimetic Desire* can best be described as unresolved. The causative mechanisms behind EDs are still mostly unknown and the scientific progress that has been made regarding, for example, genetic influence may thus far have created more of an “illusion of consensus” (Strober and Johnson 2012:158) than an actual unanimity in the field: almost everyone agrees that genes matter, but nobody knows exactly how and why.

As for EDs as historical entities, it must be noted that Girard does not deny the historicity of EDs; what he denies is the notion that only phenomena of the past have a historical quality. For Girard, the historicity of EDs is particular to the modern era. Here too, it appears that the jury is still out.

The focus on conspicuous non-consumption as part of the clinical picture is perhaps the least controversial of Girard’s ideas, although it has been criticized in ethnographic research. The contradictory nature of EDs has frequently been noted. Bruch recognized how the body of the undernourished patient becomes a conspicuous signal in itself, at once secluded and on display: “The anorexics’ very appearance announces their loneliness.” (Bruch 1973:258) Author and physician Raymond Tallis, too, notices this paradox of conspicuous non-consumption in AN: “It is rather analogous to wearing dark glasses to hide tears and so broadcasting one’s mourning.” (Tallis 2008:59) Apparently, this haunting implosion of signifying repeatedly calls for new interpretations.

However, certain parts of Girard’s exposition are problematic from a clinical point of view. Whereas he is articulate about the self-sustaining nature of mimetic processes in general, his language is somewhat inconsistent when it comes to EDs, occasionally implicating that ‘mimetic’ can be understood as ‘self-inflicted’. This is unfortunate—no matter what sparks an ED, it should be clear that the symptoms usually quickly assume a will of their own. In previous versions of the DSM, AN was depicted as a *refusal* to maintain a normal body weight; however, the wording in the DSM-5 has been altered so that ED behaviors are now described without any reference to volition (American Psychiatric Association 2013). As we have seen, a person with an ED may simultaneously experience loss of control *and* pride in rewarding aspects of the disorder, but this is not sufficient reason to depart from the otherwise predominant view of mimesis as a self-reinforcing process. An ‘agnostic’ view of causality is typically recommended: we usually do not know what causes a particular ED case and, in most cases, it does not matter, at least not in the initial stages of treatment (Lock and Le Grange 2013). This de-emphasis on personal etiology can also contribute to a loosening of the typical close identification of the patient with his or her affliction, in order to reduce self-blame and promote agency in change. Ironically, Girard does more justice to this troublesome invasiveness of EDs in *Violence and the Sacred*, where he speaks about the anthropological phenomenon of *possession*: “Indeed, how can one defend oneself against an enemy who blithely ignores all barriers between inside and outside?” (Girard 2013:186)

The Girardian model also suffers from a lack of openings towards alternative possibilities—somewhat surprisingly, given Girard’s articulation of the specific historical circumstances that have brought about the emergence of EDs. In Girard’s universe, it is very hard to picture a way out, to imagine a space for treatment and recovery. Certainly, he cannot be blamed for not offering an off-the-shelf solution to the troublesome scenario he outlines; he does, however, communicate a fateful inevitability that borders on hopelessness. If Pandora’s box has once and for all been opened and mimetic rivalry is now unleashed by the end of prohibitions, can there be any hope of escaping the maelstrom? In *Eating Disorders and Mimetic Desire*, there is no answer to this question. Unfortunately, such a gloomy outlook may feed into patients’ self-conceptualization of EDs as appropriate self-care described above, cementing ED behaviors as part of a personal identity of moral virtue.

If Girard’s view of EDs hardly opens new venues for treatment, the highlighting of mimetic processes could, however, inform the way we think about prevention. There are effective approaches in the prevention of EDs, on universal as well as specific levels (Watson et al. 2016). For universal prevention—i.e., preventive measures targeting an entire population irrespective of risk—promoting media literacy has the most support, whereas for selective prevention—i.e., preventive measures targeting a subpopulation whose risk is higher than average—there is evidence in support of so called dissonance-based interventions, whereby participants are encouraged to take a stance that is contrary to their original attitude in order to generate cognitive dissonance that promotes reflection and may lead to a shift in attitude. Both of these models of prevention fit well with the Girardian concept of mimesis in the development of EDs; however, in societies where mimesis does not necessarily entail rivalry on an individual level, these approaches may have to be modified.

A Girardian perspective could also offer rich opportunities for further research within the psychiatric field. As some commentators have noted, the discovery of *mirror neurons*—i.e., neural systems that are activated when a person observes a task being performed by somebody else, thus ‘mirroring’ the performance of the other (see for example Rizzolatti and Craigher 2004) —may shed new neuroscientific light upon Girard’s theory of mimesis (Garrels 2005). Girard also noted these implications of his theory: “If human beings suddenly ceased imitating, all forms of culture would vanish. Neurologists remind us frequently that the brain is an enormous imitating machine.” (Girard 2016:7) The neuroscientific underpinnings of mimesis in a broader context are beyond the scope of this article and to the best of my knowledge, there has been no research on a possible role of mirror neurons in the pathogenesis of EDs. Psychiatrist Jean-Michel Oughourlian, a long-time Girard collaborator, has recently tried to offer a neuropsychiatric synthesis of Girard’s concept of mimesis (Oughourlian 2016); however, he only briefly touches upon the topic of EDs.

In *Eating Disorders and Mimetic Desire*, René Girard certainly does not present an all-encompassing paradigm of EDs, weaving together the multiple strands of vulnerability at play into a coherent fabric—frankly, that would be asking a bit too much given the current state of evidence in the field. Even so, he does contribute to our clinical understanding, although perhaps not by simply adding another piece to the puzzle but rather by shifting perspectives. In my view, his take on the historicity of EDs is perhaps the most intriguing part of Girard’s account. By rejecting anachronistic ideas about EDs as a timeless entity that emerges in different shapes throughout history, Girard points instead to mimetic rivalry as the ever-emerging element and calls for a critical view of the mimetic phenomena specific to our time.

## Footnotes

1 Unless otherwise specified, all quotes in this article are from *Eating Disorders and Mimetic Desire*.

2 It should be noted that while referring to individuals as an ‘anorexic’ or a ‘bulimic’ was commonplace in the mid-1990s, this language is no longer seen as acceptable in the field: “Individuals who meet diagnostic criteria do not become the disorder (e.g., anorexics or bulimics), they remain persons, participants, or patients and our scientific writing must reflect this fact. To refer to individuals by the name of a disorder is both inaccurate and, in the case of conditions that are associated with negative social evaluation, stigmatizing.” (Weissman et al. 2016:350)
